# Asymptomatic Rotator Cuff Tendinopathy in Elderly Diabetics: Is Routine Magnetic Resonance Imaging Evaluation of the Shoulder Warranted?

**DOI:** 10.5704/MOJ.2603.004

**Published:** 2026-03

**Authors:** TP Gupta, B Sirohi, MA Jafri, S Rai

**Affiliations:** 1 Department of Orthopaedics, Cantonment General Hospital, New Delhi, India; 2 Department of Orthopaedics, Military Hospital, Agra, India; 3 Department of Orthopaedics, 166 Military hospital, Jammu Satwari, Jammu, India; 4 Department of Orthopaedics, Military Hospital, Ambala, India

**Keywords:** asymptomatic rotator cuff tendinopathy, MRI shoulder, diabetes, pericapsulitis shoulder

## Abstract

**Introduction:**

The occurrence of rotator cuff tendinopathy, which is invariably symptomatic, increases as populations age, being largely observed among patients with diabetes. The objective of the present study is to find out the occurrence of rotator cuff tendinopathy in elderly diabetic patients who were asymptomatic, composed of healthy individuals and those with diabetes mellitus.

**Materials and methods:**

The participants in this study included 87 elderly diabetic patients and 56 controls (mean age: 69.3±4.9 and 71.8±3.6, respectively), who were asymptomatic from shoulder. All patients underwent shoulder MRI examination using 1.5 tesla MRI.

**Results:**

We recorded greater tendons thickness in the diabetic patients as compared with the controls (supraspinatus tendon: 6.6±0.7mm vs 5.3±0.8mm, p<0.001; biceps tendon: 3.9±0.7mm vs 3.1±0.8mm, p<0.002). Moreover, higher incidence of supraspinatus tendon tear was noted in diabetics as compared to biceps tendon (major tears: 32 (36.7%) vs 6 (10.7%), p=0.052; minor tears: 51 (58.6%) vs 11 (19.6%), p=0.032).

**Conclusions:**

The present study suggests that age-related rotator cuff tendinopathy is more prevalent among patients with diabetes. Therefore, MRI is an investigation of choice for early detection i.e., at pre-symptomatic stages of rotator cuff tendinopathy, as the patients may develop symptoms later.

## Introduction

The incidence of rotator cuff tears in an elderly population is on an increasing trend, according to various ultrasonography (USG) studies. It varies from 0 – 15% among individuals who are 60 years of age to 30 – 50% among those who are 80 years of age. These wide differences can be explained by the various criteria used in the studies, along with the sonographic data by which the lesions were identified^1-11^.

Many studies have demonstrated that magnetic resonance imaging (MRI) is the ideal modality in detecting rotator cuff tendinopathy among asymptomatic patients as well as patients with symptoms such as painful shoulders than USG. However, the use of MRI for screening or epidemiological purposes is limited because of its high costs, especially in the context of developing countries^12-15^.

However, Ultrasound (USG) and MRI are the most commonly used imaging modalities for assessing rotator cuff pathologies. In diabetics rotator cuff tendinopathies or tears, are more common and for which USG is less sensitive and specific in detecting these pathologies and it also depends on radiologist experience. Thus, MRI is the choice of imaging for detecting early shoulder joint pathologies in asymptomatic diabetics.

The risk of developing rotator cuff tendinopathy has been reported to be greater among patients with diabetes^16-23^. Further, several authors have identified poor outcomes and restriction of shoulder movement among patients with diabetes after they underwent surgical repair^24,25^. In particular, some authors noted a higher incidence of re-tears post-surgery^26,27^. A review of the existing literature reveals that few studies have evaluated asymptomatic elderly patients with diabetes^4,5^.

USG is less sensitive and specific in detecting these pathologies and it also depends on radiologist experience. Therefore, we decided to investigate whether diabetes contributes or possesses age-related rotator cuff degeneration and whether MRI screening can be a useful tool for examining the shoulders of asymptomatic elderly patients with diabetes.

Although many studies have focused on the sonographic evaluation of supraspinatus tendon tears^1,2,21,28,29^, other rotator cuff tendons have not been studied, resulting in a gap in existing literature^30,31^. Thus, the objective of the present study is to find out the prevalence of various rotator cuff tendon conditions among asymptomatic elderly patients with diabetes.

## Materials and Methods

In a prospective cohort study conducted between January 2022 and January 2023 in a tertiary care hospital. The inclusion criteria for the study group were the following: age >65 years, diabetic patients, absence of major aches and pain in the shoulder, no injury, and absence of any gross malfunction were included. Patients with partial or complete tear of supraspinatus tendon without any pain and with full shoulder motion is also included once detected after MRI. The exclusion criteria were the following: patients with endocrinopathies, rheumatoid arthritis, autoimmune diseases such as ankylosing spondylitis and SLE, renal, hepatic, and cardiac diseases. Patients with partial or complete tears of supraspinatus tendon with pain and restriction of shoulder motion were also excluded once detected after MRI.

The criteria for selecting the control group included the following: the control group consisted of 56 individuals, matched for age and sex, but without diabetes mellitus and selected with the same inclusion/exclusion criteria. Initially 175 patients were recruited, 102 were diabetic and 73 were non-diabetic healthy elderly individuals with same age group without any shoulder symptoms. A total of 32 patients were excluded as they did not meet the inclusion criteria.

**Fig. 1:**
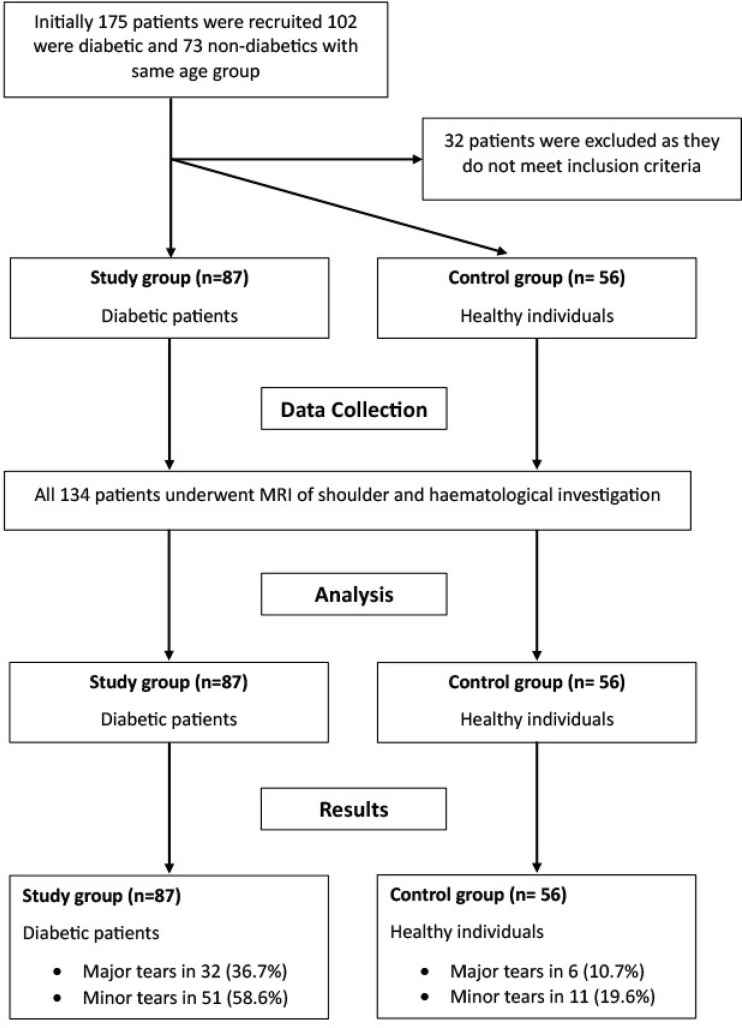
Consort flow chart and study design.

The approval for the study was taken from the ethical committee of Military Hospital Ambala India 133001, and consent was taken from all the patients. We followed the American Diabetes Association’s criteria^32^ for diagnosing non-insulin-dependent diabetes mellitus (NIDDM) in this study. The Study design has been shown in Fig. 1.

A sample size estimate using G-power software was conducted for an anticipated low proportion of 0.15 in the asymptomatic group for tear features presumed to be linked with symptoms. We aimed to identify a fourfold increase in the likelihood of symptomatic presentation (OR=4) for participants exhibiting the tear features on MRI with an alpha of 0.05 and beta=0.2. A total sample size of 134 (n1=80 and n2=54) was required to achieve a significance level of 0.05 and a power of 0.80.

The duration of the onset of diabetes was recorded, along with other associated medical conditions and medications. The participating patients were categorised based on the levels of their previous working activities. We consider desk and household work as light work, whereas heavy work includes farming, factory, and industrial work.

MRI examination was performed using standard protocol^33^. All scans were performed by the same radiologist using a 1.5 Tesla MRI (Philips, model-BV Gold Netherlands). We chose MRI over Ultrasound because it is superior with higher sensitivity and specificity and has minimal operator dependency. (a) Partial thickness tear: this is characterised by presence of a focal degeneration and partial discontinuity in the tendon, along with fluid signal intensity on T2-weighted images^33^. (b) Full-thickness tear: this is characterised by focal discontinuity in the tendon, along with a fluid signal intensity on T2-weighted images^33^.

McKean *et al*^34^, Sharma *et al*^15^ and Spencer *et al*^35^ assess the supraspinatus tendon (SST), subscapularis tendon (SBT), biceps tendon (BT), infraspinatus tendon (IST), and subacromial-subdeltoid bursa (SAD) using MRI. The BT thickness was measured in the bicipital groove while the maximal SST thickness was determined using a longitudinal view.

Zlatkin *et al*^36^ noted that the diseases affecting the rotator cuff of shoulder can be categorised into four classes. A tendon with a normal shape and T1 and T2 MRI signal intensity was considered Grade 0. A tendon with a normal shape but enhanced T1 and T2 MRI signal intensity was considered Grade I. A tendon with aberrant MRI T1 and T2 weighted signal intensity was considered Grade II.

Abnormal morphology was described as a conspicuous irregularity or thinning of the tendon. A distinct big area of discontinuity in the tendon's typical signal void was designated as a Grade III tendon. On T2WI, the discontinuity area usually displayed a stronger signal. MRI was used to estimate the Acromion-Humeral head (A-H) distance, as previously reported by McCreesh^37^ An extensive rotator cuff tear was defined as an A-H distance of less than 0.5cm, acromion stenosis as 0.5 – 1.0cm, and normal acromion as 1.0 – 1.5cm.

Using sagittal proton density imaging, the Goutallier classification was used to classify the fatty infiltration of the rotator cuff muscles^38,39^. Grade 0 denotes no fat, Grade I indicate trace fatty streaks, Grade II denotes <50% fat, Grade III represents 50% fat, and Grade IV denotes >50% fat. This grading system serves as a guide. Each patient's global fatty degeneration index (GFDI) was determined and categorised into three groups: <1, 1-1.5, and >1.540.

According to the distance between the tendon’s two ends, a tear might be minor (>1cm), large (between 1 and 3cm), or major (<3cm) as recorded by Schumaier *et al*^41^ and Lädermann *et al*^42^. For clinical purposes, we only considered minor tears (which include partial and small full-depth disruption) and major tears (which include large full depth tendon disruption).

In biceps brachii tendon ruptures, partial tears were defined hyperintensity along the tendon of the long head of the biceps in the bicipital groove suggesting bicipital tendinopathy^33,34^. Further, the involvement of SAD was determined by the presence of anechoic fluid with or without hyperintensity and synovial thickening (Hirji *et al*^43^).

We used SPSS Ver 19, and MS Excel 2019 for statistical analyses. The collected data was analysed. The percentage (%) and frequency were used for the non-continuous variables. MRI abnormalities regarding rotator cuff pathologies among diabetics were compared with controls. Finally, the thickness differences among the tendon were analysed between the right and the left shoulders, as well as according to diabetic duration. We considered p<0.05 as significant. Furthermore, the two-sample Student’s t-test and the Wilcoxon rank sum test were used to compare the variables. The associations between the categorical data were evaluated through the χ2 test.

## Results

The demographic parameters of the elderly diabetic patient group and the control group are summarised in Table I. All patients who participated in the study were similar in terms of age, sex, and activity level. It was found that medical conditions such as hypertension, coronary heart disease, and chronic kidney disease were more prevalent among patients with diabetes.

**Table I:** Demographic parameters of both groups.

Parameters	Diabetics (n=87)	Control (n=56)	P value
Age	69.3±4.9	71.8±3.6	0.762
Sex (M : F)	35 : 52	29 : 27	0.312
Hypertension	62	12	0.001
CAD	52	9	0.001
CKD	49	2	0.001
PVD	16	1	0.001
Level of physical activity			
Heavy worker	11	31	0.002
Light worker	76	25	0.003
BMI			
BMI ≥ 25	56	42	0.062
BMI < 25	31	14	0.051
HB1Ac			
< 7%	65	-	-
< 7%	32	-	-
Lipid level (Total Cholesterol)			
< 200mg	10	38	0.021
200 – 250mg	54	11	0.033
> 250mg	13	7	0.032
Smoking	21	37	0.041
Alcohol use	11	23	0.002

Abbreviations – CAD: Coronary artery disease, CKD: Chronic kidney artery disease, PVD: Peripheral vascular disease, BMI: Body mass index, HB1Ac: Hemoglobin A1c.

In the study group, 65 patients had diabetes for less than 10 years (mean 7.2±1.3), whereas 23 patients had it for more than 10 years (mean 12.1±1.7 years). Similarly, in the study group, all patients had HbA1c of less than 8.0%.

**Fig. 2:**
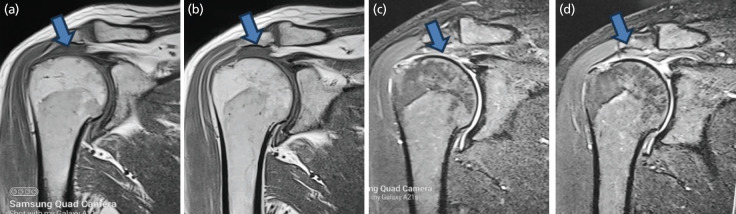
MRI images of supraspinatus tendon (SST) and biceps tendon (BT) thickness in diabetics. MRI Rt Shoulder in diabetic patients, (a and b) arrowhead showing complete tear of Supraspinatus in T1 weighted image, (c and d) arrowhead showing complete tear of Supraspinatus in T2 weighted image.

We recorded greater SST and BT thickness among patients with diabetes than in the controls, both in the right and the left shoulders (Fig. 2, Table II). In the present study, partial and complete tear considered as Major lesion, and altered MRI signal, hyperintensity of signal in rotator cuff considered as Minor lesion (Table II).

**Table II:** MRI findings regarding Rotator cuff pathologies in diabetics and control groups.

Structure	Diabetics (n=87)	Rt shoulder Control (n=56)	p	Diabetics	Lt shoulder Control	p	Diabetics	Both shoulder Control	p
**BT**									
Major lesion	6 (6.8%)	2 (3.5%)	0.482	4 (4.5%)	2 (3.5%)	1.000	10 (11.4%)	4 (7.1%)	0.566
Minor lesion	9 (10.3%)	3 (5.3%)	0.366	6 (6.8%)	2 (3.5%)	0.482	15 (17.2%)	5 (8.9%)	0.218
**SST**									
Major lesion	19 (21.83%)	3 (5.3%)	0.008	13 (14.94%)	3 (5.3%)	0.103	32 (36.7%)	6 (10.7%)	(<0.001)
Minor lesion	32 (36.78%)	7 (12.5%)	0.0019	21 (24.13%)	4 (7.1%)	0.012	51 (58.6%)	11 (19.6%)	(<0.001)
**Effusion**									
BT	27 (31.0%)	4 (7.1%)	(<0.001)	21 (24.13%)	2 (3.5%)	(<0.001)	48 (55.1%)	6 (10.7%)	(<0.001)
SAD	13 (14.9%)	3 (5.3%)	0.103	6 (6.8%)	5 (8.9%)	0.751	19 (21.8%)	8 (14.2%)	0.283
**Degeneration**									
BT	24 (27.5%)	13 (23.21%)	0.696	11(12.64%)	9(16%)	0.625	35 (40.2%)	22 (39.2%)	1.000
RC	39 (44.8%)	12 (21.42%)	0.0045	28 (32.18%)	11 (19.6%)	0.125	67 (77%)	33 (58.9%)	0.0257

Abbreviations – BT: biceps tendon, SST: supraspinatus tendon, SAD: subacromial-subdeltoid bursa RC: rotator cuff. Partial and complete tear considered as major lesion, hyperintensity of signal in rotator cuff considered as minor lesion.

**Table III:** Univariable and multivariable logistic regression analysis comparing cuff tear in diabetes.

Variables	OR (95% CI)	P-value	OR (95% CI)	P-value
Diabetes	1.43 (1.20–1.68)	<.001	1.47 (1.46, 1.52)	<.001
smoking	3.94 (3.85, 4.00)	<.001	3.91 (3.82, 3.99)	<.001
CCI	1.02 (1.02, 1.03)	<.001	0.95 (0.94, 0.96)	<.001
Age	1.00 (0.99, 1.01)	<.001	0.97 (0.96, 0.98)	<.001
Sex	1.00 (0.99, 1.01)	<.001	0.96 (0.95, 0.97)	<.001

Abbreviations – CCI: Charlson comorbidity index, CI: confidence interval, OR: odds ratio

**Fig. 3:**
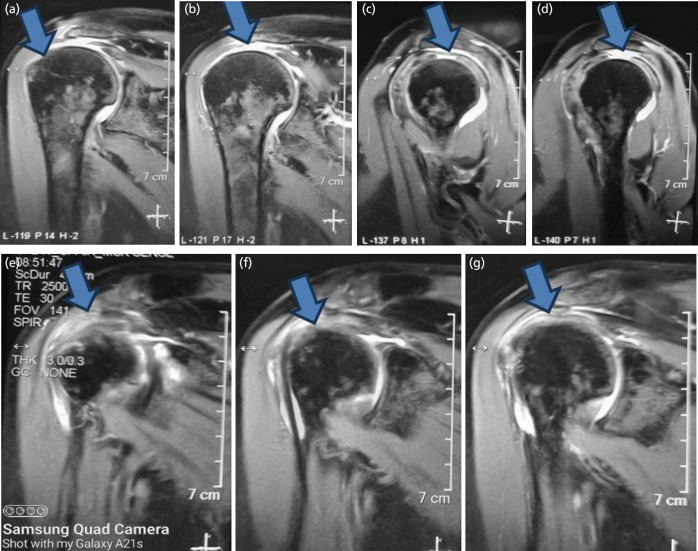
MRI images showing SAD (subacromial bursitis), tear supraspinatus and impingement of SST in diabetics. MRI Rt Shoulder in diabetic patients, (a to d) arrowhead showing complete tear of Supraspinatus in T1 weighted image, and SAD bursitis, (e to g) arrow SAD bursitis and impingement of Supraspinatus in T2 weighted image.

We also recorded greater SAD (subacromial bursitis), tear of supraspinatus tendon and impingement of SST under acromion process in diabetics than in the controls, both in the right and the left shoulders as shown in Fig. 3.

Further, among the elderly patients with diabetes, we noted a significantly higher rate of degenerative changes in the rotator cuff tendons and Biceps tendon. Similarly, the inflammatory changes and corresponding signal changes in MRI in Biceps tendon and subacromial bursitis were more frequently recorded among patients with diabetes.

In either group, SST tears were more commonly noted. Additionally, a higher occurrence of either tear (minor or major) was recorded among patients with diabetes, with massive SST tears always being associated with the involvement of IFT. Notably, we did not record any SBT tears in this study.

In the post hoc analysis using G-power, the study group and the control group were assessed, yielding a power (1–β) of 0.82 (with sample size 87 vs 56, effect size d=0.5; and error probability =0.05).

## Discussion

Several published studies have demonstrated that rotator cuff tendon tears, with or without shoulder pain and limited mobility, are more common among older diabetic patients^1-8^. It is described in many studies that periarthritis (i.e., frozen shoulder) or rotator cuff tears are more common in diabetic patients^15-18^. Furthermore, after surgical repair, diabetic patients have been found to experience reduced shoulder mobility^24,25^ and a higher incidence of re-tear^26,27^. Some researchers found that, in such cases, a poor prognosis was associated with poor quality of repaired soft tissue^24,44^.

In a study on 58 patients that were followed-up for five years, 50% of people with asymptomatic tears in an average of 2.8 years were found to experience pain and minor limitations in their shoulder functions^45,46^. Further, many authors recorded bursal and effusions around tendon and degenerative tendinopathic, because of early reactive tendinitis to minimal tendon tear following repeated microtrauma^47-50^.

We recorded a higher prevalence of rotator cuff tendinopathy in the right shoulder (i.e., the dominant side) than in the non-dominant left side of the shoulder. This may have been because of overuse, which plays a significant role in pathogenesis^7,51-53^.

In the present study, we could not quantify heavy work, light work, or their related rotator cuff tears. However, we presumed that a patient who was working at home or outside for more than six hours would be taken as a heavy worker. With this classification, we did not record any significant work-related rotator cuff tears in our study.

We noted a significantly high prevalence of rotator cuff tear tendinopathy among obese patients because obesity poses a greater risk for tear. It is in line with a previous study involving 381 patients, in which a higher rate of rotator cuff tears was observed among patients with higher BMIs than those with no tears^54^.

Furthermore, we noted that rotator cuff tendon tendinopathy was more in elderly among asymptomatic diabetic patients. These changes in the tendon were due to morphological abnormalities such as thickening and collagen disorganisation^55^. In the case of diabetes, tendon tears are more common due to mechanical weakness because of changes in biomechanics, collagen fibre re-alignment, and biochemistry, especially in TA tendon, SST, and patellar tendon under load^56^.

The duration of diabetes and rotator cuff tendinopathy have shown no significant correlation because the age of onset of diabetes is difficult to establish. Indeed, before receiving a formal diagnosis of diabetes, a patient may have mild non-insulin dependent diabetes or reduced glucose tolerance for a while.

Many authors have recorded that non-traumatic rotator cuff tears took place because of vascular factors, aging, or tendon impingement especially in subacromial space^57-59^. Further, tendon biochemical degeneration is similar among diabetic patients and aging populations. Age-related diseases and complications from diabetes are largely caused by the build-up of advanced glycation end products (AGEs)^60-62^. In fact, there is no known mechanism by which diabetes leads to onset of frozen shoulder or rotator cuff disease. Impaired microcirculation and non-enzymatic glycosylation processes may be common diabetes-related pathways and may play a role in developing the two disorders. Actually, advanced glycosylation end products (AGEs) and non-enzymatic glycosylation products are both produced in diabetics. These AGEs make collagen, tendons, and ligaments stiffer and weaker by increasing cross-linking^62^.

The non-enzymatic glycosylation of collagen with AGE development, which causes tendon swelling and rupture, is a comparable alteration. Because of these activities, glucose and its metabolic intermediates spontaneously condense with free amino groups in arginine, lysine, or hydroxylysine, which leads to the formation of AGEs.

Numerous authors have reported that AGEs alter the chemical and physical characteristics of proteins by increasing the number of cross-links between the molecules of collagen. Consequently, collagen becomes less soluble, turning harder, stiffer, weaker, and more prone to tear or rupture. It also loses its elasticity and is more susceptible to tears^63,64^. Cho *et al*^65^ conducted study on rats and observed that besides the aforementioned biochemical abnormalities the Achilles tendon alterations resembling tendinosis may result with electrical stimulation-induced repetitive, coordinated, passive, and jerky activity.

Another study noted that hypoxia and apoptosis in the rotator cuff together worsen as the macroscopic appearance of the tendon subsequently leads to tears^66^. Other authors noted that in diabetics RAGE, the vascular endothelial growth factor and cytokines are shown to be an upregulating tendency, causing a higher prevalence of tendon oedema and inflammatory tendinitis^67,68^. Furthermore, the shoulder joint also experiences the unfavourable microvascular environment brought on by hyperglycemia. The reduced circulation causes tissue hypoxia and an excess of free radicals, which may finally result in apoptosis. Joint tissue breakdown and the acceleration of degenerative processes may result from this cumulative injury.

This study has some limitations. First, the shoulder range of motion was not measured actively or passively. Second, the patients participating in the study were not followed-up to document when their symptoms first appeared.

## Conclusions

Our study shows that diabetics, especially older patients, have a higher prevalence of subclinical and asymptomatic rotator cuff lesions compared to normal individuals. How these lesions might become symptomatic over time is the subject of further research. Possible causes include poor microcirculation and non-enzymatic glycosylation processes near the synovium and tissues of the shoulder joint. Although the duration of diabetes may be linked to asymptomatic rotator cuff lesions, a direct connection between metabolic control and these lesions has not yet been studied and remains a subject for further research.

It is important to determine whether the use of specific anti-diabetic medications and/or better glycaemic management could prevent or delay the progression of rotator cuff lesions or dysfunction. MRI imaging, however, is a useful tool for detecting pre-symptomatic stages, allowing for the implementation of appropriate preventive measures.
